# Organ‐protective effect of angiotensin‐converting enzyme 2 and its effect on the prognosis of COVID‐19

**DOI:** 10.1002/jmv.25785

**Published:** 2020-04-05

**Authors:** Hao Cheng, Yan Wang, Gui‐Qiang Wang

**Affiliations:** ^1^ Department of Infectious Diseases and the Center for Liver Diseases Peking University First Hospital Beijing China; ^2^ The Collaborative Innovation Center for Diagnosis and Treatment of Infectious Diseases Zhejiang University Hangzhou Zhejiang China; ^3^ Department of Liver Diseases Peking University International Hospital Beijing China

**Keywords:** acute respiratory distress syndrome (ARDS), angiotensin‐converting enzyme 2 (ACE2), coronavirus disease 2019 (COVID‐19), renin‐angiotensin system (RAS), severe acute respiratory syndrome coronavirus 2 (SARS‐CoV‐2)

## Abstract

This article reviews the correlation between angiotensin‐converting enzyme 2 (ACE2) and severe risk factors for coronavirus disease 2019 (COVID‐19) and the possible mechanisms. ACE2 is a crucial component of the renin‐angiotensin system (RAS). The classical RAS ACE‐Ang II‐AT1R regulatory axis and the ACE2‐Ang 1‐7‐MasR counter‐regulatory axis play an essential role in maintaining homeostasis in humans. ACE2 is widely distributed in the heart, kidneys, lungs, and testes. ACE2 antagonizes the activation of the classical RAS system and protects against organ damage, protecting against hypertension, diabetes, and cardiovascular disease. Similar to SARS‐CoV, SARS‐CoV‐2 also uses the ACE2 receptor to invade human alveolar epithelial cells. Acute respiratory distress syndrome (ARDS) is a clinical high‐mortality disease, and ACE2 has a protective effect on this type of acute lung injury. Current research shows that the poor prognosis of patients with COVID‐19 is related to factors such as sex (male), age (>60 years), underlying diseases (hypertension, diabetes, and cardiovascular disease), secondary ARDS, and other relevant factors. Because of these protective effects of ACE2 on chronic underlying diseases and ARDS, the development of spike protein‐based vaccine and drugs enhancing ACE2 activity may become one of the most promising approaches for the treatment of COVID‐19 in the future.

## Research Highlights

- ACE2 plays an important role in renin‐angiotensin system and homeostasis.
- ACE2 has a protective effect on acute lung injury and for the COVID‐19 patients with underlying diseases.
- The development of spike protein‐based vaccine and drugs enhancing ACE2 activity may become one of the most promising approaches for the treatment of COVID‐19.

## 1 INTRODUCTION

**Figure 1:**
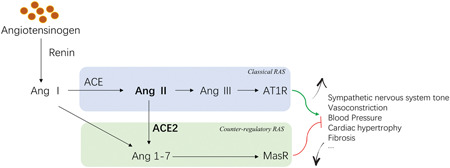
Classical and counter‐regulatory RAS. ACE, angiotensin‐converting enzyme; ACE2, angiotensin‐converting enzyme 2; Ang 1‐7, angiotensin 1‐7; Ang I, angiotensin I; Ang II, angiotensin II; Ang III, angiotensin III; AT1R, type 1 angiotensin II receptor; MasR, MAS proto‐oncogene receptor; RAS, renin‐angiotensin system

The renin‐angiotensin system (RAS) is a critical homeostasis regulation system of the human body (showed in Figure 1).^1^ The ACE‐Ang II‐AT1R pathway is called the classical RAS axis, which plays a decisive role in regulation, while the ACE2‐Ang 1‐7‐MasR‐based pathway is called the counter‐regulatory RAS axis, which plays a negative role in regulation.^2^ The primary role of the positive RAS axis is to increase sympathetic nervous system tension, cause vasoconstriction, increase blood pressure, and promote inflammation, fibrosis, and myocardial hypertrophy. The negative regulatory axis mediated by angiotensin‐converting enzyme 2 (ACE2) can antagonize these effects. In the RAS system, the same component can produce opposite physiological effects through different pathways, different components can also be linked by different pathways to have the same physiological effect. This flexibility helps the body respond quickly and coordinately to specific stimuli, from the whole body to a local area and plays an essential role in maintaining homeostasis. This paper will specifically examine the organ protection of ACE2 in the body and discuss its potential role for coronavirus disease 2019 (COVID‐19).

## 2 ANGIOTENSIN‐CONVERTING ENZYME 2

ACE2 is a homolog of ACE and was discovered in 2000.^3^ Despite the similarities between ACE and ACE2, the functions of these two enzymes are entirely different. ACE2 is active in most tissues and is widely distributed in the heart, kidney, lung, and testis.^3^, ^6^ ACE2 is widely present in human alveolar epithelial cells and small intestinal epithelial cells, as well as in arterial and venous endothelial cells and arterial smooth muscle cells. Epidermal basal cell layer of skin and basal layer of the nonkeratinized squamous epithelium of nasal, oral mucosa, and nasopharynx have ACE2 expression. ACE2 is more strongly expressed in type II epithelial cells. Glomerular tubules show ACE2 low, glomerular mesangial and glomerular endothelial cells do not express ACE2, Kupffer cells and hepatocytes, spleen, thymus, lymph nodes, bone marrow, and B and T lymphocytes and macrophages do not show ACE2.^7^, ^8^ ACE2 tissue activity is higher than its plasma activity.^9^ Its activity can not be inhibited by traditional angiotensin‐converting enzyme inhibitors (ACEIs).^3^ ACE2 has a high affinity for Ang II,^10^ and its catalytic efficiency for Ang II is 400 times greater than that for Ang I. ACE2 may show differences in different ages and sexes.^11^, ^12^

## 3 ACE2 AND ACUTE LUNG INJURY, SARS CORONAVIRUS

Lung tissue has high RAS activity and is the leading site of Ang II synthesis. Ang II is an effective pulmonary vasoconstrictor. RAS is activated during hypoxia. Ang II can not only promote the growth response of vascular smooth muscle cells but also directly promote vascular remodeling and prevent pneumonia and shunts related to lung injury.^13^ However, Ang II can also promote the occurrence of pulmonary edema and impair lung function.^14^

Acute respiratory distress syndrome (ARDS) is the most severe form of acute lung injury. It is characterized mainly by increased pulmonary vascular permeability and pulmonary edema. It is often induced by sepsis, aspiration, and pneumonia (including that caused by SARS coronavirus, bird flu, and human influenza viruses). It is a clinical, high‐death‐rate disease. ACE2 is highly expressed in the lung, and Imai et al^14^ confirmed the protective effect of ACE2 in acute lung injury. The ACE2 protein in a mouse model induced by inhaled acidic gas was significantly downregulated, while the ACE level remained stable. The inhalation of acidic gas significantly increased the levels of Ang II in the lung and plasma of wild‐type mice, and the levels of Ang II in the lung and plasma of acid‐induced ACE2 knockout (ACE2 KO) mice were further increased. Recombinant human ACE2 (rhACE2) protein treatment could reduce plasma Ang II levels and reduce acute lung injury in ACE2 KO mice and wild‐type mice. A similar phenomenon was observed in a sepsis mouse model. Studies have further demonstrated that Ang II plays a role in acute lung injury via AT1R.

ACE2 is also one of the primary receptors for SARS‐CoV invasion into the human body.^15^ What is puzzling is that SARS‐CoV infection leads to highly lethal pneumonia compared with other common cold symptoms after other coronavirus infections. The researchers found that SARS‐CoV‐infected or recombinant SARS‐spike protein‐treated wild‐type mice exhibited significantly reduced ACE2 expression in the lungs. These mice showed increased severity of pathological conditions in acute lung injury. Treating ACE2 KO mice with SARS‐spike protein did not aggravate ARDS symptoms. Therefore, the downregulation of ACE2 expression in SARS‐CoV infection may play a causal role in the pathogenesis of SARS, which provides a reasonable explanation for the progression of SARS patients into ARDS. The recent outbreak‐causing novel coronavirus pneumonia (COVID‐19) virus (2019‐nCoV, SARS‐CoV‐2) has also been shown to invade human alveolar epithelial cells through mainly ACE2.^16^ COVID‐19 ARDS patients and SARS patients have typical ARDS pathology in the lung.^17^ We believe that SARS‐CoV‐2 and SARS‐CoV may share similar pathogenesis and pathological manifestations.

## 4 RISK FACTORS FOR COVID‐19 PROGNOSIS AND ACE2

In the early outbreak of COVID‐19, epidemiological analyses of 99 patients^18^ and 138 patients^19^ showed that the average age of patients was close to 56 years and that the incidence of infections in men was higher than that of infections in women. Nearly half of the cases were complicated by chronic underlying diseases (hypertension, diabetes, cardiovascular disease, etc). A retrospective analysis of 52 cases of severely ill patients in Wuhan^20^ suggested that the severely ill patients were mainly middle‐aged and elderly individuals, two‐thirds were male, two‐thirds developed ARDS, and approximately 40% had underlying diseases. The total 28‐day case fatality rate reached 61.5%. Nanshan Zhong et al^21^ published the data of 1099 COVID patients. The patients in the severe group were older than those in the nonsevere group, and any chronic underlying diseases were more common in the severe group. An analysis of the epidemiological characteristics of the most significant sample volume of 72 314 COVID‐19 cases^22^ recently released by the Chinese Center for Disease Control and Prevention suggests that deaths occur mainly in patients over 60 years of age, with an increased mortality rate in men than in women and an increased mortality rate in patients with underlying diseases. Combining the results of existing studies, we can conclude that men over 60 years of age with chronic underlying diseases (hypertension, diabetes, cardiovascular disease, etc) and secondary ARDS carry risk factors affecting the prognosis of COVID‐19. Mortality is directly related to these risk factors.

ACE2 plays a vital role in RAS. Ang II promotes atherosclerosis in the cardiovascular system and promotes inflammation, oxidative stress, and migration of endothelial cells and vascular smooth muscle cells.^23^ ACE2 has a protective effect on many diseases with reduced expression of ACE2, such as hypertension, diabetes, and cardiovascular diseases, because it antagonizes the role of Ang II.^1^

RAS activation is an important pathophysiological mechanism of hypertension, and RAS blockers are widely used. Their antihypertensive effect comes from reducing the role of Ang II on the one hand and from the ability of ACEI and AT1R blockers (angiotensin II type 1 receptor blockers [ARBs]) to increase the circulating levels of Ang 1‐7.^24^ Studies have shown that the protective mechanism of ACE2 against hypertension is most likely achieved by the degradation of Ang II.^25^ Studies by Ferrario et al^26^ and others showed that ACE2 levels increased by 4.7 and 2.8 times when blood pressure decreased after applying ACEIs (lisinopril) and ARBs (losartan) to rats. ACE2 affects not only the development of hypertension but also potentially affects its response to treatment. In RAS‐blocked spontaneously hypertensive rats, inhibition of the vascular effects of Ang 1‐7 may reduce the antihypertensive response to these drugs.^24^ Animal experiments have found that Ang 1‐7 infusion treatment can significantly improve vascular endothelial function and inhibit atherosclerotic lesion development in Ang 1‐7 transgenic apolipoprotein E knockout (ApoE KO) mice receiving atherogenic high‐fat diets.^27^ Studies by Thomas et al^28^ provided direct evidence for the occurrence and development of ACE2 in atherosclerotic plaques. It was also found that the RAS blockade could prevent atherosclerosis in ApoE/ACE2 double knockout mice. This study provided the basis for ACEI combined with ACE2 treatment to reduce atherosclerosis. ACE2 can also antagonize cardiac fibrosis and ventricular remodeling caused by the long‐term effects of Ang II. Wysocki et al,^29^ Kassiri et al,^30^ and others provided strong evidence for the direct roles of ACE2 and Ang 1‐7 in ventricular remodeling expression and regulation. In diabetic nephropathy, downregulation of renal tubular ACE2 is associated with proteinuria and tubular damage,^31^ and further inhibition of ACE2 aggravates renal damage.^32^ rhACE2 can reduce blood pressure while reducing renal damage.^33^

As ACE2 provides a pathway for SARS‐CoV‐2 to invade the body, it increases the chance of viral infection. However, ACEIs do not directly affect ACE2 activity. The use of ACEIs to instead increase ACE2 activity has been shown in animal experiments. This mixed‐use enhances cardiovascular protection. Therefore, we do not consider it appropriate for patients with COVID‐19 to discontinue long‐term RAS blockers.^34^, ^35^ To determine whether ACEI/ARB drugs can be used in patients who lack traditional indications, prospective controlled studies are needed to provide evidence.

COVID‐19 prognosis is related to age and sex. The expression of ACE2 decreases with increasing age. ACE2 expression is higher in young people than in elderly individuals and higher in females than in males.^11^, ^12^ This pattern does not match the characteristic of severely ill COVID‐19 patients being mostly elderly males. We believe that whether the level of ACE2 expression is high or low is not a key factor affecting the prognosis of patients with COVID‐19. The relationship between sex and prognosis requires additional data to verify.

**Figure 2:**
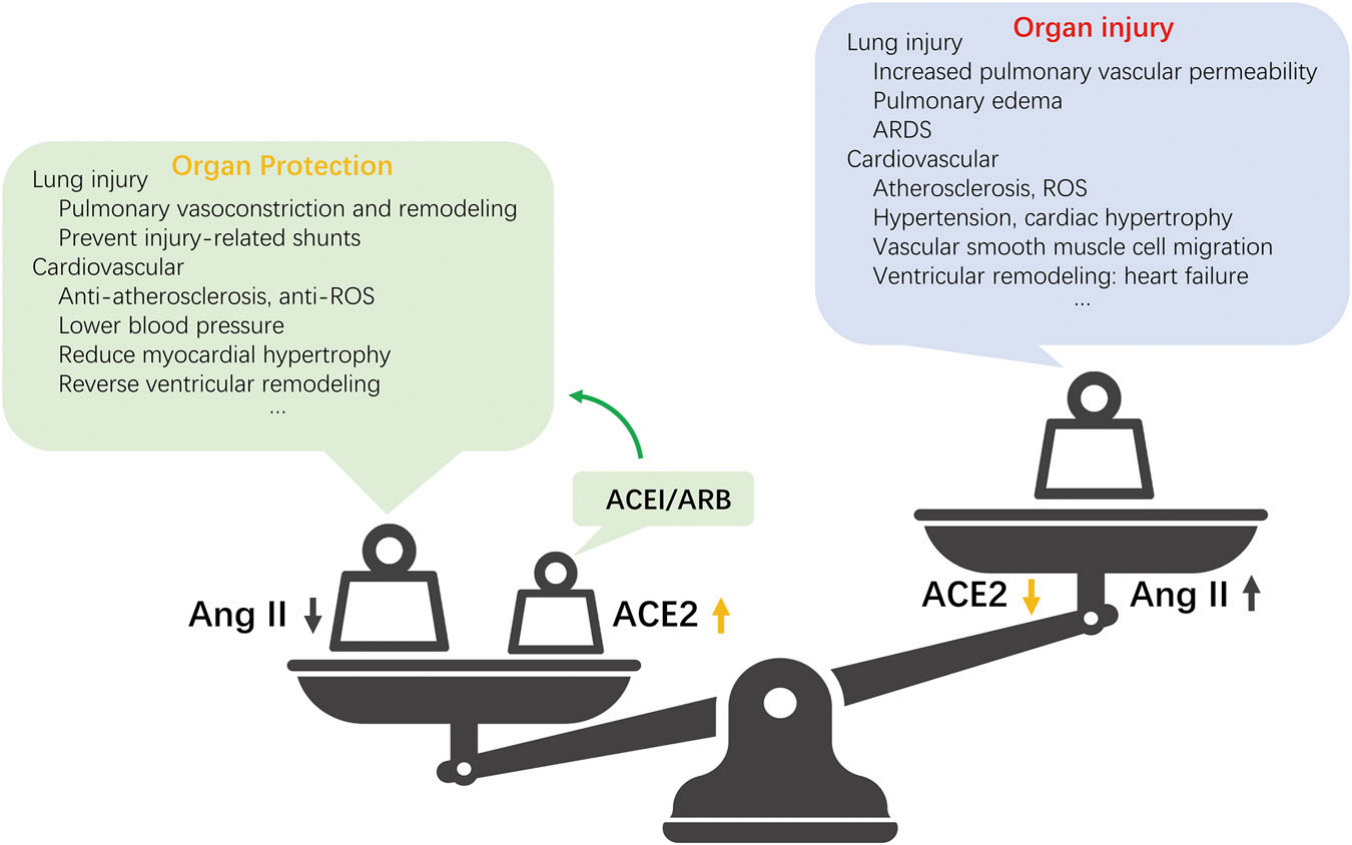
The role of ACE2 in organ protection. ACEI, angiotensin‐converting enzyme inhibitors; ACE2, angiotensin‐converting enzyme 2; Ang II, angiotensin II; ARB, type 1 angiotensin II receptor; ARDS, acute respiratory distress syndrome; ROS, reactive oxygen species

The prognosis of severely ill patients with COVID‐19 may be related to the decrease in ACE2 activity in elderly patients with chronic underlying diseases. SARS‐CoV‐2 infection reduces ACE2 activity and receptor consumption, further exacerbating pathophysiological mechanisms, such as Ang II/ACE2 regulation imbalance (showed in Figure 2). There are several potential approaches to address ACE2‐mediated COVID‐19, such as spike protein‐based vaccine (rely on the fact that ACE2 is the COVID‐19 receptor), inhibition of transmembrane protease activity (essential for entry through interaction with ACE2 receptor), blocking ACE2 receptor, and delivering the soluble form of ACE2. Many studies have been conducted to explore the therapeutic potential of ACE2. Most likely the most clinically significant potential is the cardioprotective effect of rhACE2 itself, which has been proven, as it can further enhance the vascular protective effect in patients using ACEI or ARB drugs.^1^, ^36^ Forty‐four patients^37^ with ARDS were well tolerated after using rhACE2, and they are most likely to represent the first clinical application in the field of ARDS.

## 5 CONCLUSION

ACE2 is an essential part of the RAS, and it has extensive vascular and organ protection functions in hypertension, diabetes, cardiovascular disease, and ARDS. Similar to SARS‐CoV, SARS‐CoV‐2 also invades the human body through ACE2. According to existing research, men over 60 years old with chronic underlying diseases (hypertension, diabetes, cardiovascular disease, etc) and secondary ARDS carry risk factors affecting the prognosis of COVID‐19. There is currently no effective drug for the treatment of COVID‐19, and we speculate that ACE2 spike protein‐based vaccine and rhACE2 may become one of the most promising approaches for future treatment and improve the prognosis of patients with COVID‐19.

## CONFLICT OF INTERESTS

The authors declare that there are no conflict of interests.

## AUTHOR CONTRIBUTIONS

CH wrote the article, including the concept of this article, the definition of intellectual content, and data acquisition. YW and G‐QW designed and reviewed the manuscript for its intellectual content.
